# Supplementary material for: Zooplankton impact on lipid biomarkers in water column vs. surface sediments of the stratified Eastern Gotland Basin (Central Baltic Sea)
Source: PLoS One. 2020 Jun 12;15(6):e0234110. doi: 10.1371/journal.pone.0234110 (PMC7292411; doi:10.1371/journal.pone.0234110)
Supplement: S1 Table — Only compounds making up > 1% of the total FA in at least one of the samples are shown. Numbers denote carbon numbers (= chain length) of n-FA and the number of double bonds, respectively (e.g. 18:1 represents n-octadecenoic acid). Numbers in superscript refer to the position and configuration of double bonds; roman numbers in superscript refer to minor isomers whose double bond positions have not been determined; ai-15 refers to 12-methyltetradecanoic acid (anteiso-pentadecanoic acid). Bars illustrate the relative abundances of individual compounds in a given fraction. No entry: compound not detected, or present in very low amounts (i.e., not quantified). *Only relative abundances (in % of the total) are available for the Filter (60–95 m) sample (values given in italics). (PDF) [file pone.0234110.s001.pdf]

FA [µg g^{-1} C_{org}]	Phytoplankton (0-25 m)		Zooplankton (25-60 m)		Filter* (60-95 m)		Zooplankton (60-90 m)	
	NL	PLFA	NL	PLFA	NL	PLFA	NL	PLFA
14:0	636	819	821	233	*5.1*	*4.2*	1031	40
15:0	135	38	308	37	*3.3*	*3.3*	108	9
ai- 15	67	26	95	25	*1.1*	*0.8*	202	4
16:0	3493	8138	7453	1902	*30.4*	*39.2*	8335	467
$16:1^{I}$	391	152		19	*9.7*		608	7
$16:1^{\omega7c}$	550	1227	993	98	*0.9*	*3.6*	8737	56
$16:1^{II}$	42	36	105	23	*0.1*	*0.1*	700	9
16:3	53	281						
17:0	122	91	263	91	*1.2*	*1.4*		10
18:0	895	476	1132	227	*21.6*	*32.6*	1029	66
$18:1^{\omega9c}$	1544	193	9717	474	*13.9*	*2.4*	123002	660
$18:1^{\omega7c}$	415	323	1720	281	*1.1*	*1.5*	2557	61
$18:1^{II}$		16	209	43			1418	108
$18:2^{\omega6c}$	990	1178	5191	197	*2.8*	*0.7*	21741	136
$18:3^{\omega6}$	61	130	166	6	*0.2*		508	
$18:3^{\omega3}$	1607	4724	5466	155		*0.5*		21
$18:3^{I}$		2016		36		*0.2*		16
18:4	722		2933		*1.1*		10370	
20:0	38	21		5	*1.4*			
$20:1^{\omega9}$	107		646	78	*0.3*	*0.4*	1134	18
20:2	57		485				384	
$20:3^{I}$	52	40	472	6	*0.2*		3947	8
$20:5^{\omega3}$	1418	788	8448	161	*0.9*	*0.4*	23014	115
21:0		11	14	193	*0.1*	*0.2*		2
$22:6^{\omega3}$	2688	1662	15755	6	*1.1*	*0.4*	26483	98
$24:1^{\omega9}$	57	63	817	70		*0.1*	241	18
total	16140	22449	63208	4365			235551	1930
