# Supplementary material for: Zooplankton impact on lipid biomarkers in water column vs. surface sediments of the stratified Eastern Gotland Basin (Central Baltic Sea)
Source: PLoS One. 2020 Jun 12;15(6):e0234110. doi: 10.1371/journal.pone.0234110 (PMC7292411; doi:10.1371/journal.pone.0234110)
Supplement: S2 Table — Only compounds making up > 1% of the total FA in at least one of the samples are shown. Numbers denote carbon numbers (= chain length) of n-FA and the number of double bonds, respectively (e.g. 18:1 represents n-octadecenoic acid). Numbers in superscript refer to the position and configuration of double bonds; roman numbers in superscript refer to minor isomers whose double bond positions have not been determined; i-15, ai-15, and i-16 refer to 13- and 12-methyltetradecanoic acid (iso- and anteiso-pentadecanoic acid) and 14-methylpentadecanoic (iso-hexadecanoic acid), respectively. Bars illustrate the relative abundances of individual compounds in a given sample. No entry: compound not detected, or present in very low amounts (i.e., not quantified). Note that no separation was made between NL and PLFA for the sedimentary lipids. (PDF) [file pone.0234110.s002.pdf]

| FA [µg $g^{-1}$ $C_{org}$] | 0-1 cm | 1-2 cm | 3-4 cm | 4-5 cm | 5-6 cm | 6-7 cm | 7-8 cm | 8-9 cm | 10-12 cm |
|---|---|---|---|---|---|---|---|---|---|
| 14:0 | 2236 | 2419 | 1472 | 289 | 638 | 390 | 290 | 92 | 49 |
| *i-15* | 285 | 207 | 120 | 39 | 53 | 37 | 28 | 15 | 5 |
| *ai-15* | 246 | 210 | 201 | 83 | 122 | 80 | 61 | 35 | 10 |
| 15:0 | 91 | 74 | 58 | 29 | 37 | 31 | 21 | 17 | 5 |
| 16:0 | 4457 | 4918 | 3318 | 1497 | 1476 | 827 | 648 | 471 | 277 |
| *i-16* | | | | | 37.69 | 25.63 | 23.33 | 8.69 | 4.54 |
| $16:1^{\omega 7c}$ | 991 | 1023 | 388 | 98 | 123 | 122 | 76 | 48 | 33 |
| $16:1^{II}$ | 382 | 322 | | | 29 | 24 | 14 | | |
| $18:3^{\omega 6}$ | 167 | | | | | | | | |
| $18:2^{\omega 6c}$ | 304 | 269 | 113 | | 54 | 38 | | | |
| $18:1^{\omega 9c}$ | 4571 | 4894 | 961 | 263 | 211 | 130 | 80 | 64 | 38 |
| $18:1^{\omega 7c}$ | 381 | 315 | 77 | 51 | 43 | 23 | 25 | 15 | 5 |
| 18:0 | 454 | 460 | 550 | 215 | 192 | 125 | 147 | 91 | 46 |
| 20:0 | 325 | 279 | 520 | 235 | 243 | 139 | 96 | 89 | 83 |
| 21:0 | 27 | | 74 | 42 | 30 | 11 | 9 | 11 | 10 |
| $22:6^{\omega 3}$ | 328 | 106 | 66 | | 19 | 11 | | | 2 |
| 22:0 | 689 | 588 | 1167 | 462 | 458 | 286 | 184 | 163 | 157 |
| 23:0 | 20 | | | 19 | 27 | 18 | 16 | 15 | 12 |
| 24:0 | 163 | 119 | 297 | 229 | 276 | 176 | 153 | 134 | 91 |
| 25:0 | 23 | | | | 32 | 24 | 22 | 19 | 13 |
| 26:0 | 359 | 185 | 323 | 203 | 328 | 304 | 218 | 194 | 109 |
| 27:0 | 45 | | | | 57 | 51 | 39 | 30 | |
| 28:0 | | | 411 | 190 | 257 | 219 | 181 | 168 | 110 |
| total | 16542 | 16389 | 10116 | 3943 | 4743 | 3091 | 2331 | 1680 | 1060 |
