# Supplementary material for: Zooplankton impact on lipid biomarkers in water column vs. surface sediments of the stratified Eastern Gotland Basin (Central Baltic Sea)
Source: PLoS One. 2020 Jun 12;15(6):e0234110. doi: 10.1371/journal.pone.0234110 (PMC7292411; doi:10.1371/journal.pone.0234110)
Supplement: S3 Table — Bars illustrate the relative abundances of individual compounds in a given sample. No entry: compound not detected, or present in very low amounts (i.e., not quantified). *Only relative abundances (in % of the total) are available for the Filter (60–95 m) sample (values given in italics). (PDF) [file pone.0234110.s003.pdf]

Sterols [µg g^{-1} C_{org}]	Phytoplankton (0-25 m)	Zooplankton (25-60 m)	Filter* (60-95 m)	Zooplankton (60-90 m)
24-Norcholesta-5,22-dien-3β-ol	137	1116		
Cholesta-5,22-dien-3β-ol	303	2286	*1.1*	273
Cholest-5-en-3β-ol (cholesterol)	3245	10223	*84.4*	1466
24-Methylcholesta-5,22E-dien-3β-ol	195	979		
24-Ethylcholesta-5,22E-dien-3β-ol	157	95	*1.3*	
24-Ethylcholest-5-en-3β-ol (sitosterol)	176	258	*3.8*	
total	4212	14957		1738
