# Supplementary material for: Zooplankton impact on lipid biomarkers in water column vs. surface sediments of the stratified Eastern Gotland Basin (Central Baltic Sea)
Source: PLoS One. 2020 Jun 12;15(6):e0234110. doi: 10.1371/journal.pone.0234110 (PMC7292411; doi:10.1371/journal.pone.0234110)
Supplement: S4 Table — Bars illustrate the relative abundances of individual compounds in a given sample. No entry: compound not detected, or present in very low amounts (i.e., not quantified). Concentrations of tetrahymanol are given for comparison. (PDF) [file pone.0234110.s004.pdf]

Compounds [µg g^{-1} C_{org}]	0-1 cm	1-2 cm	3-4 cm	4-5 cm	5-6 cm	6-7 cm	7-8 cm	8-9 cm	10-12 cm
Cholesta-5,22-dien-3β-ol	175	70	23	38	46	30	22	19	
Cholest-5-en-3β-ol (cholesterol)	828	435	366	339	291	227	118	117	57
5α-Cholestan-3β-ol (cholestanol)	588	367	224	193	259	230	193	173	111
24-Methylcholesta-5,22E-dien-3β-ol	124	82	53	32	50	36	29	16	15
24-Methyl-5α-cholest-22E-en-3β-ol	48	52	48	27	37	33	20	15	
24-Methylcholest-5-en-3β-ol	62								
24-Methyl-5α-cholestan-3β-ol	89								
24-Ethylcholesta-5,22E-dien-3β-ol	229	112	78	48	38	53	40	26	15
24-Ethylcholest-5-en-3β-ol (sitosterol)	630	253	344	390	315	263	205	146	81
24-Ethyl-5α-cholestan-3β-ol	163	76	82	92	104	76	81	81	30
4α,23,24-Trimethyl-5α-cholest-22E-en-3β-ol (dinosterol)	701	334	630	574	171	243	143	171	168
total									
Stanol/stenol ratio (C_{27})	0.71	0.84	0.61	0.57	0.89	1.01	1.63	1.48	1.95
Stanol/stenol ratio (C_{29})	0.26	0.30	0.24	0.23	0.33	0.29	0.40	0.55	0.37
Tetrahymanol	183	103	188	152	110	108	84	67	38
