# Supplementary material for: Zooplankton impact on lipid biomarkers in water column vs. surface sediments of the stratified Eastern Gotland Basin (Central Baltic Sea)
Source: PLoS One. 2020 Jun 12;15(6):e0234110. doi: 10.1371/journal.pone.0234110 (PMC7292411; doi:10.1371/journal.pone.0234110)
Supplement: S5 Table — Only compounds making up > 1% of the total (acyclic) alcohols in at least one of the samples are shown. Numbers denote carbon numbers (= chain length) of n-alcohols and the number of double bonds, respectively (e.g. 18:1 represents n-octadecen-1-ol). Roman numbers in superscript refer to further isomers whose double bond positions have not been determined; i-15 and ai-17 refer to 13-methyltetradecan-1-ol (iso-pentadecan-1-ol) and 15-methylhexadecan-1-ol (anteiso-heptadecan-1-ol), respectively. Bars illustrate the relative abundances of individual compounds in a given sample. No entry: compound not detected, or present in very low amounts (i.e., not quantified). *Only relative abundances (in % of the total) are available for the Filter (60–95 m) sample (values given in italics). (PDF) [file pone.0234110.s005.pdf]

Alcohol [µg g^{-1} C_{org}]	Phytoplankton (0-25 m)	Zooplankton (25-60 m)	Filter* (60-95 m)	Zooplankton (60-90 m)
12:0	10		*0.9*	51
14:0	108	2684	*8.7*	46857
15:0		88	*2.1*	994
i-15		303		1049
16:0	240	7403	*23.2*	91107
17:0		59	*0.7*	274
ai-17		52	*2.3*	300
18:0	100	262	*28.5*	4313
$18:1^{I}$		706		5831
$18:1^{II}$		460		6862
$18:1^{III}$				193
19:0			*1.2*	
20:0	46		*6.1*	
$20:1^{I}$	57		*5.5*	
$20:1^{II}$		412		3348
ai-21	10		*2.3*	
22:0			*3.1*	
22:1		89	*1.7*	759
24:0	103		*4.3*	
24:1		215	*1.2*	1260
26:0			*1.1*	
total	673	12732		163199
