# Supplementary material for: Zooplankton impact on lipid biomarkers in water column vs. surface sediments of the stratified Eastern Gotland Basin (Central Baltic Sea)
Source: PLoS One. 2020 Jun 12;15(6):e0234110. doi: 10.1371/journal.pone.0234110 (PMC7292411; doi:10.1371/journal.pone.0234110)
Supplement: S6 Table — Only compounds making up > 1% of the respective fraction in at least one of the samples are shown. Numbers denote carbon numbers (= chain length) of n-alcohols and numbers of double bonds (i.e., all compounds shown in the table are saturated). Bars illustrate the relative abundances of individual compounds in a given sample. No entry: compound not detected, or present in very low amounts (i.e., not quantified). (PDF) [file pone.0234110.s006.pdf]

| Alcohol [µg g$^{-1}$ $C_{org}$] | 0-1 cm | 1-2 cm | 3-4 cm | 4-5 cm | 5-6 cm | 6-7 cm | 7-8 cm | 8-9 cm | 10-12 cm |
|---|---|---|---|---|---|---|---|---|---|
| 14:0 | 41 | | | | 9 | | 3 | | |
| 15:0 | | | | | | | | | |
| 16:0 | 165 | 62 | | 27 | 42 | 27 | 18 | 15 | 5 |
| 17:0 | | | | | 10 | | | | |
| 18:0 | 33 | | | 22 | 21 | 12 | 12 | 15 | 5 |
| 20:0 | 59 | | | 53 | 49 | 28 | 19 | 29 | 14 |
| 21:0 | | | | 72 | 57 | 30 | 12 | 28 | |
| 22:0 | 522 | 514 | 2107 | 1615 | 1092 | 321 | 205 | 349 | 548 |
| 23:0 | 18 | | | 40 | 43 | 22 | 13 | 19 | 12 |
| 24:0 | 169 | 136 | 538 | 490 | 431 | 182 | 150 | 195 | 235 |
| 25:0 | 8 | | | | 20 | 16 | 11 | 11 | 5 |
| 26:0 | 240 | | 163 | 154 | 209 | 151 | 119 | 122 | 75 |
| 27:0 | 8 | | | | 8 | 11 | 8 | 7 | |
| 28:0 | 121 | | 128 | 81 | 114 | 109 | 75 | 66 | 54 |
| total | 1385 | 713 | 2936 | 2554 | 2106 | 909 | 644 | 855 | 953 |
