# Supplementary material for: Zooplankton impact on lipid biomarkers in water column vs. surface sediments of the stratified Eastern Gotland Basin (Central Baltic Sea)
Source: PLoS One. 2020 Jun 12;15(6):e0234110. doi: 10.1371/journal.pone.0234110 (PMC7292411; doi:10.1371/journal.pone.0234110)
Supplement: S1 Text — (PDF) [file pone.0234110.s007.pdf]

Untreated sample was loaded on a fast-heating Pt-filament of a Pyrola 2000 device (Pyrolab SB) coupled to a Varian CP3800 GC and a Varian 1200L MS. The filament was heated in 2 ms from 200 to 360 °C (hold 30 s). The gas chromatograph was equipped with a Phenomenex Zebron ZB-5 capillary column (30 m, 0.1 μm film thickness, inner diameter 0.25 mm), and used helium (1.7 ml/min) as the carrier gas. Pyrolysis products were flushed onto the GC column at an injector temperature of 300 °C, and a split rate of 20. The GC oven temperature was ramped from 40 (3 min) to 310 °C at 10 °C min−1, and was held for 15 min. Electron-ionization mass spectra were recorded at 70 eV in full-scan mode (mass range 50–450, scan time 0.35 s).
